# Understanding activity trends in electrochemical water oxidation to form hydrogen peroxide

**DOI:** 10.1038/s41467-017-00585-6

**Published:** 2017-09-26

**Authors:** Xinjian Shi, Samira Siahrostami, Guo-Ling Li, Yirui Zhang, Pongkarn Chakthranont, Felix Studt, Thomas F. Jaramillo, Xiaolin Zheng, Jens K. Nørskov

**Affiliations:** 1 SUNCAT Center for Interface Science and Catalysis, Department of Chemical Engineering, Stanford University, 443 Via Ortega, Stanford, CA 94305 USA; 2 Department of Mechanical Engineering, Stanford University, Stanford, CA 94305 USA; 3 SUNCAT Center for Interface Science and Catalysis, SLAC National Accelerator Laboratory, Menlo Park, CA, 94025 USA; 4 School of Physics and Engineering, Henan University of Science and Technology, Luoyang, 471023 China; 5 Department of Mechanical Engineering, Tsinghua University, Beijing, 100084 China; 6 Institute of Catalysis Research and Technology, Karlsruhe Institute of Technology, 76344 Eggenstein-Leopoldshafen, Germany; 7 Institute for Chemical Technology and Polymer Chemistry, Karlsruhe Institute of Technology, 76131 Karlsruhe, Germany

## Abstract

Electrochemical production of hydrogen peroxide (H_2_O_2_) from water oxidation could provide a very attractive route to locally produce a chemically valuable product from an abundant resource. Herein using density functional theory calculations, we predict trends in activity for water oxidation towards H_2_O_2_ evolution on four different metal oxides, i.e., WO_3_, SnO_2_, TiO_2_ and BiVO_4_. The density functional theory predicted trend for H_2_O_2_ evolution is further confirmed by our experimental measurements. Moreover, we identify that BiVO_4_ has the best H_2_O_2_ generation amount of those oxides and can achieve a Faraday efficiency of about 98% for H_2_O_2_ production.

## Abstract (web summary)

Producing hydrogen peroxide via electrochemical oxidation of water is an attractive route to this valuable product. Here the authors theoretically and experimentally investigate hydrogen peroxide production activity trends for a range of metal oxides and identify the optimal bias ranges for high Faraday efficiencies.

## Introduction

Hydrogen peroxide (H_2_O_2_) is an important chemical with a wide range of applications in industry, such as paper and textile manufacturing and environmental protection for detoxification and color removal of wastewater^1^. Currently, H_2_O_2_ is produced indirectly via the anthraquinone oxidation process, which is an energy-demanding multi-electron process and requires large plants^1^. Moreover, transportation of H_2_O_2_ to the place of use adds additional challenges due to safety concerns. Broader usage of H_2_O_2_ could benefit from the capability of direct on-site production. Electrochemical synthesis of H_2_O_2_ provides a straightforward route for on-site production and ideally solves the issues associated with the indirect anthraquinone route^2–9^. One of the attractive possible routes for electrochemical H_2_O_2_ generation is via two-electron oxidation of water (Eq. 1)^10–12^:

1 $$2{{\rm{H}}_2}{\rm{O}} \to {{\rm{H}}_2}{{\rm{O}}_2} + 2\left( {{{\rm{H}}^ + } + {{{e}}^ - }} \right)\quad E^\circ = 1.76\,{\rm{V}}$$

This process (Eq. 2) is desirable since it can be coupled with hydrogen evolution reaction (Eq. 2) to simultaneously produce two valuable products: H_2_O_2_ and H_2_ (Eq. 3) in a single electrochemical device using only water as raw material. Such a device can also be coupled with photoabsorbers to utilize sunlight for both reactions:^12–14^

2 $$2\left( {{{\rm{H}}^ + } + {{{e}}^ - }} \right) \to {{\rm{H}}_2}\quad E^\circ = 0.0\,{\rm{V}}$$

3 $$2{{\rm{H}}_2}{\rm{O}} \to {{\rm{H}}_2}{{\rm{O}}_2} + {{\rm{H}}_2}$$

However, the two-electron water oxidation (Eq. 1) must compete with the four-electron oxidation reaction for O_2_ generation (Eq. 4) and the one-electron oxidation reaction for producing OH radical (Eq. 5)^12^.

4 $$2{{\rm{H}}_2}{\rm{O}} \to {{\rm{O}}_2} + 4\left( {{{\rm{H}}^ + } + {{{e}}^ - }} \right)\quad E^\circ = 1.23\,{\rm{V}}$$

5 $${{\rm{H}}_2}{\rm{O}} \to {\rm{O}}{{\rm{H}}^ \bullet }\left( {{\rm{aq}}} \right) + \left( {{{\rm{H}}^ + } + {{{e}}^ - }} \right)\quad E^\circ = 2.38\,{\rm{V}}$$

The relevant intermediates of the one- (Eq. 5 ^12^), two- (Eq. 1) and four-electron (Eq. 4) water oxidation reactions are OH*, O* and OOH*. Historically, the O_2_ generation reaction (Eq. 4) has been the main focus of the water oxidation research^15–29^ and less attention has been paid to the selective two-electron oxidation reaction of water to H_2_O_2_ (Eq. 1), which is considered as a much more difficult process. A main challenge in realizing such a photoelectrochemical production of H_2_O_2_ system is to find a material that can selectively and efficiently produce H_2_O_2_ from water. Electrochemical oxidation of water has been reported over various metal oxides, such as MnO_*x*_^10, 11^, WO_3_-BiVO_4_^14, 30, 31^ and TiO_2_^32–36^. These pioneering works have not only demonstrated the potential of H_2_O_2_ production over metal oxides but also suggested that BiVO_4_ is the best oxide for H_2_O_2_ production^14, 31^. Nevertheless, little is known about the energy barrier and the limiting potential for H_2_O_2_ generation for different metal oxides. Even less is known about how the H_2_O_2_ generation efficiency of these metal oxides vary with applied biases both theoretically and experimentally. Such study on the bias-dependent H_2_O_2_ production is critical for understanding the competition among one-, two- and four-electron pathways under different bias, a prerequisite for identifying the optical bias range for H_2_O_2_ generation.

In the present work, we theoretically investigate the activity trends of four different oxides (i.e., WO_3,_ SnO_2_, TiO_2_ and BiVO_4_) towards water oxidation for H_2_O_2_ production with further experimental validation. Both the calculated and measured onset potentials for H_2_O_2_ production increase in the sequence of WO_3_, BiVO_4_, SnO_2_ and TiO_2_. Among all these four oxides, BiVO_4_ is identified as the best catalyst for the two-electron water oxidation in dark and under illumination, and this result is consistent with previous studies on comparing different metal oxides for H_2_O_2_ production^14, 31^. Importantly, we identify the optimal bias range for BiVO_4_ to produce H_2_O_2_ in dark (~2.9–3.3 V vs RHE) and under illumination (~1.7 V–2.3 V vs RHE). As such, BiVO_4_ achieves a high faraday efficiency (FE) of 70% in dark and 98% under 1 sun illumination.

## Results

### Theoretical analyses

**Fig. 1:**
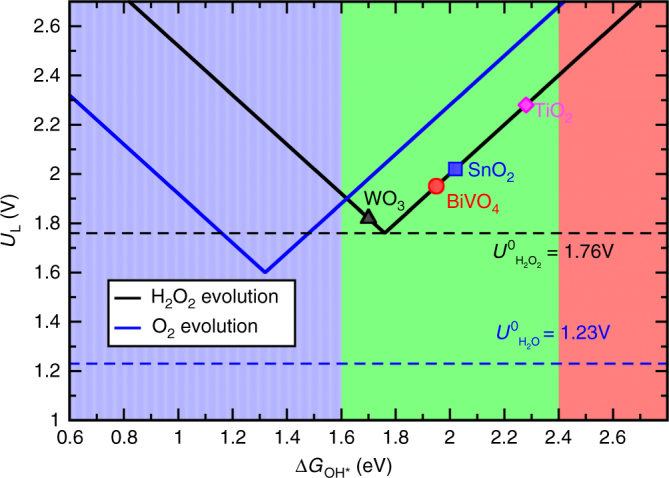
Activity volcano plots. It is based on calculated limiting potentials as a function of calculated adsorption energies of OH* ($\Delta {G_{{\rm{O}}{{\rm{H}}^{\rm{*}}}}}$) for the two-electron oxidation of water to hydrogen peroxide evolution (*black*) and the four-electron oxidation to oxygen evolution (*blue*). The corresponding equilibrium potentials for each reaction have been shown in *dashed lines*

As discussed above, H_2_O_2_ synthesis from water oxidation is a challenging reaction. This is due to the fact that selectivity and activity of the materials are largely limited by several criteria imposed by the thermodynamics of the competing reactions^13^. The adsorption free energies of relevant intermediates of the one- (Eq. 5), two- (Eq. 1) and four-electron (Eq. 4) water oxidation reactions, i.e., OH*, O* and OOH* can be calculated using density functional theory (DFT). We show here that the free energies of OH* and O* are key parameters determining the selectivity and activity towards different oxidation products, O_2_ (Eq. 4), OH radical (Eq. 5) or H_2_O_2_ (Eq. 1)^13^. Using DFT, we calculated the free energies of OH*, O* and OOH* on BiVO_4_ (details of calculations in Supplementary Note 1). We only focus on the (111) surface, which has been shown theoretically and experimentally to be stable and exposed in the BiVO_4_ crystal structure^37^. In addition, we have taken the OH*, O* and OOH* free energies for WO_3_(100), TiO_2_(110) and SnO_2_(110) from reported DFT calculations (Supplementary Note 1, Supplementary Fig. 1 and Supplementary Tables 1, 2)^13, 38–40^. We use the computational hydrogen electrode (CHE) model, which exploits that the chemical potential of a proton–electron pair is equal to gas-phase H_2_ at standard conditions. The electrode potential is taken into account by shifting the electron energy by *–eU*, where *e* and *U* are the elementary charge and the electrode potential, respectively^41^. The limiting potential for the electrochemical reaction to occur is defined as the lowest potential, at which all the reaction steps are downhill in free energy following the previous report^13^. Figure 1 shows the activity volcano plots based on the calculated limiting potentials as a function of the calculated free energy of OH* $\left( {\Delta {G_{{\rm{O}}{{\rm{H}}^{\rm{*}}}}}} \right)$ for both two-electron (black) and four-electron (blue) oxidation reactions. The corresponding equilibrium potentials for each reaction have been shown in dashed lines.

From the thermodynamic point of view, materials with strong OH adsorption energy (shaded in blue in Fig. 1) will further oxidize OH* to O* and OOH*, following the complete four-electron oxidation reaction (Eq. 4) to evolve oxygen. Therefore, electrocatalysts with weak OH* free energy will have low selectivity towards the four-electron pathway but high preference towards the two-electron route. At the same time, the OH* free energy should be strong enough to dissociate the water molecule and provide a good thermodynamic driving force, for the two-electron pathway towards H_2_O_2_. The free energy for H_2_O_2_ formation is ~3.5 eV, twice of the equilibrium potential for Eq. 1, so the electrocatalyst should have $\Delta {G_{{\rm{O*}}}}$ ≳ $3.5\,{\rm{eV}}$. Given the fact that the *O and *OH energies are generally found to scale ($\Delta {G_{{\rm{O*}}}} = 2\Delta {G_{{\rm{OH*}}}} + 0.28$)^38^, this sets a lower limit for OH* free energy, i.e., $\Delta {G_{{\rm{OH*}}}}$ ≳ $\frac{{3.5}}{2} - \frac{{0.28}}{2} \sim 1.6{\kern 1pt} \,{\rm{eV}}$. The upper limit for $\Delta {G_{{\rm{OH}}}}$ is set by the free energy of OH radical formation in the solution (Eq. 5), since too weak OH* free energy with Δ*G*_OH*_ > ~2.4 eV drives the reaction towards OH radical formation (pink shaded area in Fig. 1). Hence, the combined thermodynamic criteria and scaling relation indicates a selective catalyst for H_2_O_2_ evolution should have $\Delta {G_{{\rm{OH*}}}}$ from ~1.6 to 2.4 eV. This thermodynamic analysis suggests that WO_3_, SnO_2_, BiVO_4_ and TiO_2_ should be able to generate H_2_O_2_ within certain values of the OH* free energy (shaded in green in Fig. 1). To increase the selectivity region for H_2_O_2_ evolution, we need to identify catalyst materials that largely deviate from the O* and OH* scaling relation^42^.

Aside from the high selectivity, the two-electron oxidation reaction (Eq. 1) ideally should also have high activity with low overpotential. The theoretical overpotential is defined as the difference between the limiting potential and equilibrium potential (1.76 V for the two-electron path). The overpotential is governed by the binding of OH* to the catalyst surface, so controlling the overpotential is a matter of tuning the free energy of OH*^41^. An OH* free energy ($\Delta {G_{{\rm{OH*}}}}$) of 1.76 eV, when calculated at zero potential and relative to liquid water, will give zero overpotential. The calculated theoretical limiting potential for BiVO_4_ is 1.95 V, hence it has a theoretical overpotential of ~0.2 V for the two-electron oxidation reaction. The activity of BiVO_4_ can be further improved with different doped metals such as Sr and Ru (Supplementary Note 2, Supplementary Fig. 2 and Supplementary Table 3). The calculated limiting potentials for WO_3_, SnO_2_ and TiO_2_ are 1.82, 2.02 and 2.27 V, respectively. In the following, we show that the trend in theoretical limiting potentials for WO_3_, BiVO_4_, SnO_2_ and TiO_2_ is in very good agreement with experimental measurements.

### Materials fabrication

Experimentally, we evaluate the H_2_O_2_ evolution performance of four oxides: WO_3_, BiVO_4_, SnO_2_ and TiO_2_ by determining their onset potentials, faraday efficiencies and production rates of H_2_O_2_ per geometric area of electrodes. All the oxides were synthesized on transparent and conductive fluorine-doped tin oxide (FTO) substrates. The WO_3_ was synthesized by flame vapor deposition (FVD). BiVO_4_, SnO_2_, and TiO_2_ were synthesized by a sol–gel process (see Methods and Supplementary Note 3). Since the electrochemical performance of each oxide is affected by its loading, each oxide film was individually optimized to yield the highest FE for the dark electrochemical measurement before taken for comparison. For example, Supplementary Fig. 3 shows that the measured FE for H_2_O_2_ production varies with the BiVO_4_ loading, which was controlled by varying the precursor concentration during the spin coating process (Supplementary Fig. 4). The low loading results in low BiVO_4_ coverage and exposed FTO, leading to a low H_2_O_2_ production. On the other hand, the high coverage results in high film resistance that impedes the charge transport process. Hence, for each oxide investigated, its loading on FTO was individually optimized for H_2_O_2_ production and the dependence of the FE on sample preparation conditions for each oxide is listed in Supplementary Table 4.

### H_2_O_2_ production comparison under dark conditions

**Fig. 2:**
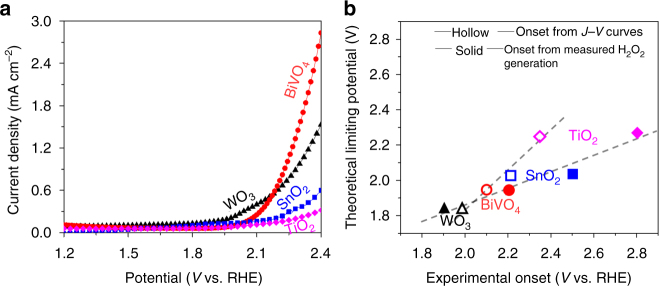
*J–V* curves and onset potentials. **a** *J–V* curves of four metal oxides without illumination, for which the current onset suggest the onset of H_2_O_2_ production. **b** Theoretical predicted onset potentials vs experimental measured onset potentials for H_2_O_2_ production. The values on the *y*-axis are the theoretical limiting potential obtained from Fig. 1. As to the *x*-axis, the values of hollow points were the potentials for the *J–V* curves of different metal oxides to reach 0.2 mA cm^−2^ in Fig. 2a, while the values of the solid points were the potentials at which the generated H_2_O_2_ concentration reaches 1 ppm, by measuring a 1 cm^2^ sample in the 20 ml electrolyte for 10 min

Next, we measured the current–voltage (*J–V*) curves for the four oxides (WO_3_, BiVO_4_, SnO_2_ and TiO_2_) without illumination (Fig. 2a). The *J–V* onset, as well as the experimental measured H_2_O_2_ evolution onset potentials (defined as the potential at which the H_2_O_2_ concentration reaches 1 ppm), are compared with the calculated theoretical potentials in Fig. 2b. It can be seen that both the measured current onset potential (hollow symbols) and the onset potential for H_2_O_2_ generation (solid symbols) increase in the order of WO_3_ < BiVO_4_ < SnO_2_ < TiO_2_, which agrees with the theoretical predication, supporting the validity of using $\Delta {G_{{\rm{OH*}}}}$ as a descriptor to analyze the H_2_O_2_ evolution onset (Fig. 1). The measured onset potentials are higher than the calculated values, which is likely due to the additional kinetic barriers to be overcome in the actual experiments.

**Fig. 3:**
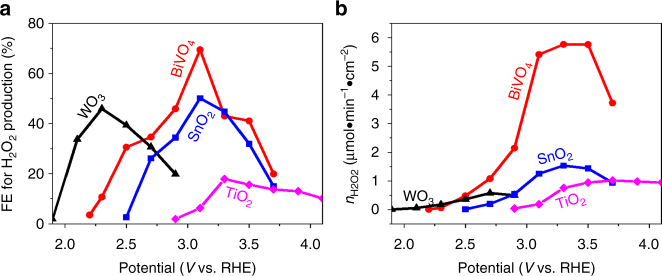
The faraday efficiency (FE) and mole amount of H_2_O_2_ under dark. **a** The FE and **b** the mole amount (*n*) of H_2_O_2_ generation (*n*) vs potential (*V*) without illumination. Both show that BiVO_4_ has the highest FE and *n* for H_2_O_2_ production over other metal oxides

We further quantified the FE and the amount of H_2_O_2_ generated from the oxides as functions of the applied biases. As shown in Fig. 3a, all the metal oxides investigated share the similar FE trend with increasing applied bias: the FE first increases to a maximum value and then decreases. Or equivalently, for each metal oxide, there is an optimal potential window for H_2_O_2_ production, due to the competition with one-electron and four-electron oxidation reactions as predicted by theory (Fig. 1). Among all the oxides investigated, BiVO_4_ achieves the highest FE of 70% at 3.1 V vs RHE. In addition, BiVO_4_ also has the highest H_2_O_2_ production rate per geometric area of electrode (Fig. 3b), due to the combined high FE (Fig. 3a) and high current density (Fig. 2a).

### H_2_O_2_ production on BiVO_4_ under different conditions

**Fig. 4:**
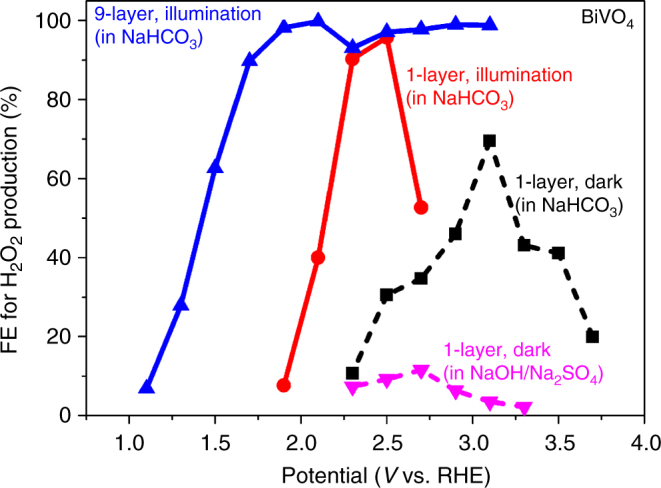
The FE for BiVO_4_ under both dark and illumination. FE vs applied bias for BiVO_4_ under different conditions, including different electrolytes in darkness, same electrolyte between darkness and illumination, and different layers under illumination. The thicker BiVO_4_ with light illumination in NaHCO_3_ shows best performance for H_2_O_2_ production

The above results clearly indicate that BiVO_4_, among the four oxides investigated, has the best electrochemical properties towards H_2_O_2_ production, which is consistent with the previous screening studied of different metal oxides for H_2_O_2_ generation^31^. Next, we further investigated the influence of other conditions on the H_2_O_2_ production of using BiVO_4_, including the electrolyte, light illumination and sample thickness. The two dash lines in Figure 4 are the measured FEs for the thin 1-layer BiVO_4_ (i.e., spin coating once) under dark using two different electrolytes: 1 M NaHCO_3_ (pH = 8.3) and 0.5 M Na_2_SO_4_ (adjusted to pH = 8.3 with NaOH). Clearly, NaHCO_3_ is a much better electrolyte than NaOH/Na_2_SO_4_ for the H_2_O_2_ production from water oxidation. This observation is consistent with recent work by Fuku et al.,^14^ which reported that HCO_3_^–^ is beneficial for the H_2_O_2_ production. In addition, as shown in Fig. 4, illumination not only shifts the onset potential to lower values but also increases the FEs for H_2_O_2_ production from 70% to over 95% for the 1-layer BiVO_4_ in NaHCO_3_. The reasons are that illumination introduces additional photogenerated charge carriers for H_2_O_2_ production and supplies photovoltage to allow H_2_O_2_ generation from water oxidation at a lower external bias^43^. To further enhance the benefits under illumination, we increase the loading of BiVO_4_ by spin coating nine times (referred as 9-layer BiVO_4_) to enhance the light absorption. The increased thickness for BiVO_4_ further shifts the onset potential for H_2_O_2_ to less than 1.1 V vs RHE, which is over 1.1 V lower than that of the dark conditions. This lowered onset potential and increased FEs strongly support that BiVO_4_ is a promising photoanode material for the H_2_O_2_ production in a photoelectrochemical system. The *J–V* curve and the measured H_2_O_2_ generation rate under illumination for this 9-layer BiVO_4_ are shown in Supplementary Fig. 5. In addition, we have measured the evolved gaseous O_2_ and H_2_ and the measured FEs are shows in Supplementary Fig. 6. The figure shows that the sum of the FE (O_2_) and FE (H_2_O_2_) is about 98–103%, confirming the accuracy in our H_2_O_2_ concentration measurement. Finally, the photoelectrochemical stability of BiVO_4_ is known to be an issue when the electrolyte is far from neutral conditions because the V^5+^ tends to dissolve into solution^44, 45^. However, we used the bicarbonate electrolyte with a measured pH value of 8.3; hence, BiVO_4_ is relatively stable in this near neutral region^46^.

## Discussion

In the present work, we utilized DFT calculations in conjunction with experimental measurements to study the activity of two-electron water oxidation towards H_2_O_2_ evolution over four metal oxides, namely WO_3_, BiVO_4_, SnO_2_ and TiO_2_. Both the calculated and measured onset potentials for H_2_O_2_ production increase in the sequence of WO_3_, BiVO_4_, SnO_2_ and TiO_2_. Among all these four oxides, BiVO_4_ produces the highest faraday efficiency (~70%) and largest amount for H_2_O_2_ under dark. The peak faraday efficiency BiVO_4_ is further increased to 98% by adding illumination, optimizing electrolyte and optimizing the thickness of BiVO_4_. Those optimizations also lower the onset potential from 2.2 V to <1.1 V. These results suggest that BiVO_4_ is an excellent photoanode candidate for electrochemical and photoelectrochemical H_2_O_2_ production. The theoretical simulation and experimental demonstration illustrated in this work have furthered the understanding of the activity and selectivity of water oxidation to H_2_O_2_ on metal oxide surfaces. Our result has opened an avenue for novel photoelectrochemical device designs with fundamental mechanism study that utilize solar energy and water to produce an oxidative product with higher value beyond O_2_.

## Methods

### Fabrication of various metal oxides on FTO

The BiVO_4_ precursor solution was prepared from a mixture of bismuth nitrate hexahydrate (BiN_3_O_9_·5H_2_O, 99.99%; Aldrich) and vanadyl acetylacetonate (C_10_H_14_O_5_V, 98%; Aldrich), which were added to a solution of acetylacetone (C_5_H_8_O_2_; Aldrich) and acetic acid (CH_3_COOH, 99.70%; Fisher) with a ratio of 1:0.12, followed by sonication for 10 min. After sonication, a dark green solution was obtained and the solution is usually used within a day after the preparation. For the one named 1-layer, the mole concentration of Bi was varied from 0.08 M, and 0.04 M, 0.02 M, to 0.01 M. For a typical spin coating, 100 µl of the BiVO_4_ precursor solution was dropped on a pre-cleaned FTO glass followed by spin coating (500 r.p.m. for 5 s and 1500 r.p.m. for 30 s). The samples were then annealed at 100 °C for 10 min, and 300, 400, and 500 °C for 5 min at each temperature. Similar step-wise annealing process was commonly used for metal oxide fabrication, and the purpose is to slowly evaporate the solvent to achieve a better film morphology. For spin coating multiple layers, the same process above was repeated for multiple times. Finally, the coated FTO was annealed in a box furnace at 500 °C for 2 h.

SnO_2_ was fabricated by using a sol–gel process similar with BiVO_4_. Firstly the precursor solution was made by dissolving 0.1932 g tin chloride in 10 ml 2-methoxyethanol (CH_3_OCH_2_CH_2_OH, 99.8%; Aldrich) and 0.2 ml acetylacetone (CH_3_COCH_2_COCH_3_, ≥99.3%; Aldrich) as the best condition, and sonicated for 30 min. After then the solution was put in a fume hood with aging for one night. The solution was spin coated on top of cleaned FTO with first 5 s, 500 r.p.m. and second 35 s, 2000 r.p.m. steps. Annealing process was carried out by using step by step method (100 °C for 5 min, 300 °C for 5 min and finally 455 °C for 30 min).

TiO_2_ was fabricated from a paste making and coating process. About 9.5 ml ethanol and 0.5 ml water were mixed and 0.5 g polyethylene glycol (Aldrich) was added and the mixture was sonicated for 30 min. Then 0.25 g TiO_2_ powder (Aldrich) as the best condition was added and the suspension was sonicated for another 10 min. Then the suspension was put on a hot plate and heated at 120 °C until the total volume reached 5 ml to get the TiO_2_ paste. The paste was used to spin coat or blade coat on top of FTO glass, followed by the annealing process at 400 °C for 2 h.

WO_3_ was synthesized by FVD method^46^, for which a W wire (0.5 mm in diameter; Aldrich) was oxidized by flame as the WO*x* vapor source. Those WO*x* vapor further deposited on FTO as W_18_O_49_ nanowires. The optimized FVD condition followed the one used in Rao et al.’s work^46^, with 18.4SLPM:12.5SLPM CH_4_ to air flow ratio and a substrate temperature 550 °C for 10 min of deposition (Supplementary Table 4). W_18_O_49_ nanowires were further converted to WO_3_ nanowires by annealing at 500 °C in a box furnace for 1 h.

### Characterizations

The H_2_O_2_ production from various anodes was detected by using the potentiostat with the three-electrode system, in which the Ag/AgCl electrode was used as a reference electrode and Ti foil as the counter electrode. Silver paste and Teflon tape were used to make metal contact and to define area when necessary. The measurements under illumination were obtained under 1 Sun at AM 1.5 G. The amount of generated H_2_O_2_ was detected by using the standard H_2_O_2_ strips (Indigo Instruments). In addition, the generated H_2_O_2_ concentration was further confirmed with a titration process by using potassium permanganate (KMnO_4_, ≥99.0%; Aldrich). The permanganate ion has a dark purple color, and the color disappears during titration when the MnO_4_^−^ is totally consumed based on the following equation:

6 $$2{\rm{MnO}}_4^ - + 5{{\rm{H}}_2}{{\rm{O}}_2} + 6{{\rm{H}}^ + } \to 2{\rm{M}}{{\rm{n}}^{2 + }} + 5{{\rm{O}}_2} + 8{{\rm{H}}_2}{\rm{O}}$$

In this work, the sulfuric acid (H_2_SO_4_; Acros Organics) was used as the H^+^ source. We measured five H_2_O_2_ solution samples with different degrees of dilution from a same initial concentration 100 ppm, by both the standard strips and the permanganate titration methods. The results are shown in Supplementary Fig. 7, which shows that two methods basically agree with each other, confirming the accuracy of the H_2_O_2_ concentration measurement.

To make the result of the onset potential of H_2_O_2_ generation value more fair and accurate, in addition to the dark current onset here it was also defined as the potential at which the H_2_O_2_ generation starts to be detected (beyond 1 ppm), by measuring a 1 cm^2^ sample in a 20 ml electrolyte for 10 min. H_2_ and O_2_ were detected by the gas chromatography analysis, and the morphology of the samples were obtained using scanning electron microscopy (FEI XL30 Sirion SEM). The FE for H_2_O_2_ production (%) is calculated by

7 $${\rm{FE}} = \frac{{{\rm{Amount}}\,{\rm{of}}\,{\rm{generated}}\,{{\rm{H}}_2}{{\rm{O}}_2}\left( {{\rm{mol}}} \right)}}{{{\rm{theoretical}}\,{\rm{generated}}\,{{\rm{H}}_2}{{\rm{O}}_2}}} \times 100$$

where the theoretical amount of H_2_O_2_ is equal to the total number of electrons divided by two (in mol). The FE of H_2_O_2_ is calculated based on the accumulated amount of H_2_O_2_ after the 10 min measurement for each condition. The FEs for H_2_ and O_2_ are calculated in a similar way, in which the theoretical amount of H_2_ is also equal to the total amount of electrons divided by two (in mol), while the theoretical amount of O_2_ is equal to the total amount of electrons divided by four (in mol), respectively.

### Computational details

Density functional theory calculations are done using the projector-augmented wave method and a plane-wave basis set as implemented in the Vienna Ab Initio Simulation Package (VASP). The valence configurations are treated as 6*s* ^2^6*p* ^3^ for Bi, 3*d* ^3^4*s* ^2^ for V, 2*s* ^2^2*p* ^4^ for O and 1*s* ^1^ for H. The cutoff energy for plane-wave basis functions is 400 eV. The bulk and surface properties of BiVO_4_ are optimized within GGA-PBE. For a more accurate description, the calculations are done within GGA-rPBE for the adsorption energies of OH*, O* and OOH* species on the BiVO_4_(111) surface. The reference energies of the pristine slab, H_2_, H_2_O and O_2_ molecules are also carefully treated within GGA-rPBE. For periodic slab calculations, slabs of six metal-oxygen layers are separated by at least 12 Å of vacuum. The atomic positions within the top two layers of the slabs were allowed to relax with the force convergence of 0.02 eV per Å. Spin polarization is considered in all the calculations.

### Data availability

Data supporting the findings of this study are available within the article and its supplementary information files, and from the corresponding author upon reasonable request.

## Electronic supplementary material

Supplementary Information

Peer Review File

## References

[1] Campos-Martin JM, Blanco-Brieva G, Fierro JLG (2006). Hydrogen peroxide synthesis: an outlook beyond the anthraquinone process. Angew. Chem. Int. Ed. Engl..

[2] Siahrostami S (2013). Enabling direct H_2_O_2_ production through rational electrocatalyst design. Nat. Mater..

[3] Verdaguer-Casadevall A (2014). Trends in the electrochemical synthesis of H_2_O_2_: enhancing activity and selectivity by electrocatalytic site engineering. Nano Lett..

[4] Fellinger T-P, Hasché F, Strasser P, Antonietti M (2012). Mesoporous nitrogen-doped carbon for the electrocatalytic synthesis of hydrogen peroxide. J. Am. Chem. Soc..

[5] Choi CH (2014). Hydrogen peroxide synthesis via enhanced two-electron oxygen reduction pathway on carbon-coated Pt surface. J. Phys. Chem. C.

[6] Park J, Nabae Y, Hayakawa T, Kakimoto M (2014). Highly selective two-electron oxygen reduction catalyzed by mesoporous nitrogen-doped carbon. ACS Catal..

[7] Choi CH (2016). Tuning selectivity of electrochemical reactions by atomically dispersed platinum catalyst. Nat. Commun..

[8] Mase K, Yoneda M, Yamada Y, Fukuzumi S, Klug DR (2016). Seawater usable for production and consumption of hydrogen peroxide as a solar fuel. Nat. Commun..

[9] Mase K, Yoneda M, Yamada Y, Fukuzumi S (2016). Efficient photocatalytic production of hydrogen peroxide from water and dioxygen with bismuth vanadate and a Cobalt(II) chlorin complex. ACS Energy Lett.

[10] Izgorodin A, Izgorodina E, MacFarlane DR (2012). Low overpotential water oxidation to hydrogen peroxide on a MnOx catalyst. Energy Environ. Sci..

[11] McDonnell-Worth C, MacFarlane DR (2014). Ion effects in water oxidation to hydrogen peroxide. RSC Adv.

[12] Bard, A. J., Roger, P., Hordan, J. *Standard Potentials in Aqueous Solution* (M. Dekker, 1985).

[13] Viswanathan V, Hansen HA, Nørskov JK (2015). Selective electrochemical generation of hydrogen peroxide from water oxidation. J. Phys. Chem. Lett..

[14] Fuku K (2016). Efficient oxidative hydrogen peroxide production and accumulation in photoelectrochemical water splitting using a tungsten trioxide/bismuth vanadate photoanode. Chem. Commun..

[15] Burke MS, Enman LJ, Batchellor AS, Zou S, Boettcher SW (2015). Oxygen evolution reaction electrocatalysis on transition metal oxides and (Oxy)hydroxides: activity trends and design principles. Chem. Mater..

[16] Reier T, Oezaslan M, Strasser P (2012). Electrocatalytic oxygen evolution reaction (OER) on Ru, Ir, and Pt catalysts: a comparative study of nanoparticles and bulk materials. ACS Catal.

[17] Cheng Y, Jiang SP (2015). Advances in electrocatalysts for oxygen evolution reaction of water electrolysis-from metal oxides to carbon nanotubes. Prog. Nat. Sci. Mater. Int.

[18] Diaz-Morales O, Ledezma-Yanez I, Koper MTM, Calle-Vallejo F (2015). Guidelines for the rational design of Ni-based double hydroxide electrocatalysts for the oxygen evolution reaction. ACS Catal.

[19] Fabbri E, Habereder A, Waltar K, Kötz R, Schmidt TJ (2014). Developments and perspectives of oxide-based catalysts for the oxygen evolution reaction. Catal. Sci. Technol.

[20] Gong M, Dai H (2014). A mini review of NiFe-based materials as highly active oxygen evolution reaction electrocatalysts. Nano Res.

[21] Diaz-Morales O (2016). Iridium-based double perovskites for efficient water oxidation in acid media. Nat. Commun..

[22] Lee Y, Suntivich J, May KJ, Perry EE, Shao-Horn Y (2012). Synthesis and activities of rutile IrO _2_ and RuO _2_ nanoparticles for oxygen evolution in acid and alkaline solutions. J. Phys. Chem. Lett..

[23] Dionigi F, Strasser P (2016). NiFe-based (oxy)hydroxide catalysts for oxygen evolution reaction in non-acidic electrolytes. Adv. Energy Mater.

[24] Reier T, Nong HN, Teschner D, Schlögl R, Strasser P (2016). Electrocatalytic oxygen evolution reaction in acidic environments - reaction mechanisms and catalysts. Adv. Energy Mater.

[25] Spoeri C, Kwan JTH, Bonakdarpour A, Wilkinson D, Strasser P (2017). The stability challenges of oxygen evolving catalysts: Towards a common fundamental understanding and mitigation of catalyst degradation. Angew. Chem..

[26] Han B (2015). Activity and stability trends of perovskite oxides for oxygen evolution catalysis at neutral pH. Phys. Chem. Chem. Phys..

[27] Hong WT, Welsch RE, Shao-Horn Y (2016). Descriptors of oxygen-evolution activity for oxides: a statistical evaluation. J. Phys. Chem. C.

[28] Zou S (2015). Fe (oxy)hydroxide oxygen evolution reaction electrocatalysis: intrinsic activity and the roles of electrical conductivity, substrate, and dissolution. Chem. Mater..

[29] Burke MS (2015). Revised oxygen evolution reaction activity trends for first-row transition-metal (oxy)hydroxides in alkaline media. J. Phys. Chem. Lett..

[30] Fuku K (2017). Photoelectrochemical hydrogen peroxide production from water on a WO3/BiVO4 photoanode and from O_2_ on an Au cathode without external bias. Chem. Asian J..

[31] Fuku K, Miyase Y, Miseki Y, Gunji T, Sayama K (2016). Enhanced oxidative hydrogen peroxide production on conducting glass anodes modified with metal oxides. ChemistrySelect.

[32] GOTO H (2004). Quantitative analysis of superoxide ion and hydrogen peroxide produced from molecular oxygen on photoirradiated TiO2 particles. J. Catal..

[33] Hirakawa T, Yawata K, Nosaka Y (2007). Photocatalytic reactivity for O_2_− and OH radical formation in anatase and rutile TiO_2_ suspension as the effect of H_2_O_2_ addition. Appl. Catal. A Gen.

[34] Cai R, Kubota Y, Fujishima A (2003). Effect of copper ions on the formation of hydrogen peroxide from photocatalytic titanium dioxide particles. J. Catal..

[35] Zhang J, Nosaka Y (2013). Quantitative detection of OH radicals for investigating the reaction mechanism of various visible-light TiO 2 photocatalysts in aqueous suspension. J. Phys. Chem. C.

[36] Sánchez-Quiles D, Tovar-Sánchez A (2014). Sunscreens as a source of hydrogen peroxide production in coastal waters. Environ. Sci. Technol..

[37] Li G-L (2017). First-principles investigation of the surface properties of fergusonite-type monoclinic BiVO _4_ photocatalyst. RSC Adv.

[38] Man IC (2011). Universality in oxygen evolution electrocatalysis on oxide surfaces. ChemCatChem.

[39] Siahrostami S, Björketun ME, Strasser P, Greeley J, Rossmeisl J (2013). Tandem cathode for proton exchange membrane fuel cells. Phys. Chem. Chem. Phys..

[40] Montoya JH, Garcia-Mota M, Nørskov JK, Vojvodic A (2015). Theoretical evaluation of the surface electrochemistry of perovskites with promising photon absorption properties for solar water splitting. Phys. Chem. Chem. Phys..

[41] Nørskov JK (2004). Origin of the overpotential for oxygen reduction at a fuel-cell cathode. J. Phys. Chem. B.

[42] Siahrostami S, Li GL, Viswanathan V, Nørskov JK (2017). One- or two-electron water oxidation, hydroxyl radical, or H2O2 evolution. J. Phys. Chem. Lett..

[43] Walsh A, Yan Y, Huda MN, Al-Jassim MM, Wei S-H (2009). Band edge electronic structure of BiVO_4_: elucidating the role of the Bi s and V d orbitals. Chem. Mater..

[44] Sayama K (2003). Photoelectrochemical decomposition of water on nanocrystalline BiVO4 film electrodes under visible light. Chem. Commun..

[45] Rozhkova, E. A. & Ariga, K. (eds) *From Molecules to Materials: Pathways to Artificial Photosynthesis* (Springer International Publishing, 2016).

[46] Rao PM (2014). Simultaneously efficient light absorption and charge separation in WO_3_ /BiVO_4_ Core/Shell nanowire photoanode for photoelectrochemical water oxidation. Nano Lett..

