# Supplementary material for: Understanding activity trends in electrochemical water oxidation to form hydrogen peroxide
Source: Nat Commun. 2017 Sep 26;8:701. doi: 10.1038/s41467-017-00585-6 (PMC5615073; doi:10.1038/s41467-017-00585-6)
Supplement: Supplementary file 1 — Supplementary Information [file 41467_2017_585_MOESM1_ESM.pdf]

Description of Supplementary Files

File Name: Supplementary Information
Description: Supplementary Figures, Supplementary Tables, Supplementary Notes and Supplementary References

File Name: Peer Review File

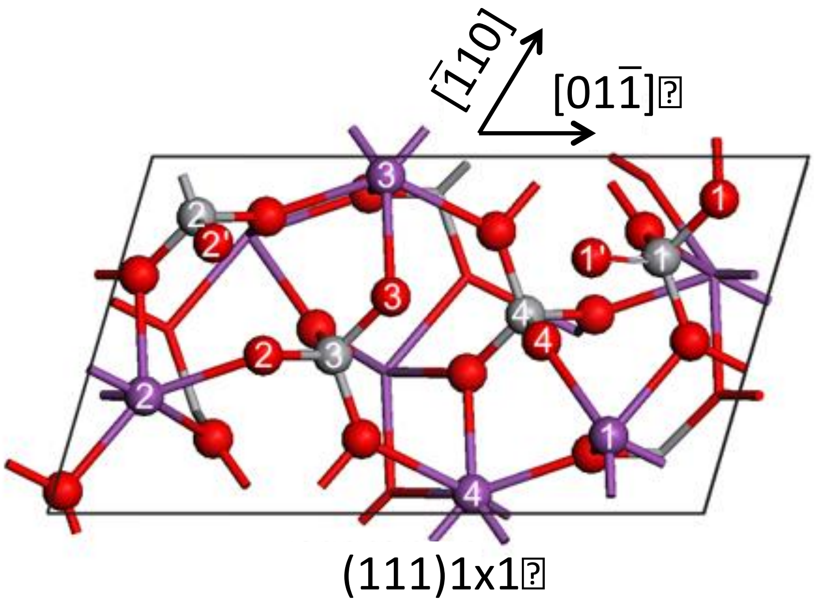

Supplementary Figure 1. Top view of $BiVO_4$ (111) 1x1 cell. Atoms underneath surface BiO_n ($5 \le n \le 7$) and VO_4 polyhedra are denoted by sticks. Adsorption of OH*, O* and OOH* occurs on the bridge sites between Bi1 and Bi4 as well as between Bi2 and Bi3.

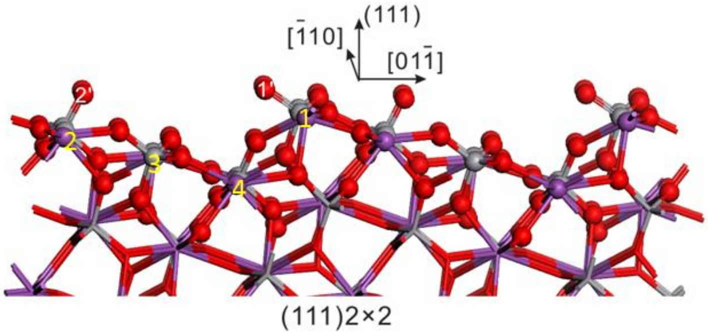

Supplementary Figure 2. Doping effect. Different studied Bi sites for substituting with doped metals are displayed in the $BiVO_4$ (111).

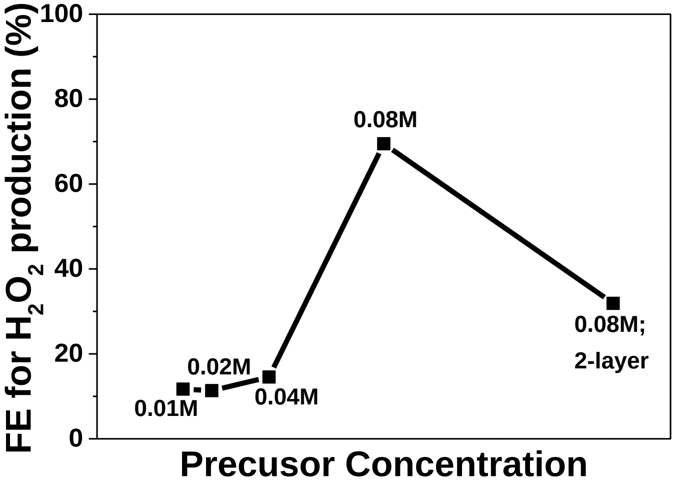

Supplementary Figure 3. Faraday efficiency (FE) in terms of precursor concentration. for H_2O_2 production as a function of precursor concentration: The material used here is $BiVO_4$, and the precursor was made from a mixture of bismuth nitrate hexahydrate ($BiN_3O_9{\cdot}5H_2O$, 99.99%, Aldrich), and vanadyl acetylacetonate ($C_{10}H_{14}O_5V$, 98%, Aldrich), which were added to a solution of acetylacetone ($C_5H_8O_2$, Aldrich) and acetic acid (CH_3COOH, 99.70%, Fisher) with a ratio of 1:0.12. The concentration value shown in the figure is for Bi. The FE was measured @3.1V vs. RHE under dark condition in 1M $NaHCO_3$, pH 8.3. From the figure it can be seen the 0.08M one shows the highest FE value compared to other precursor concentrations ones, as well as the one with same concentration but spin coated twice (named 2-layer).

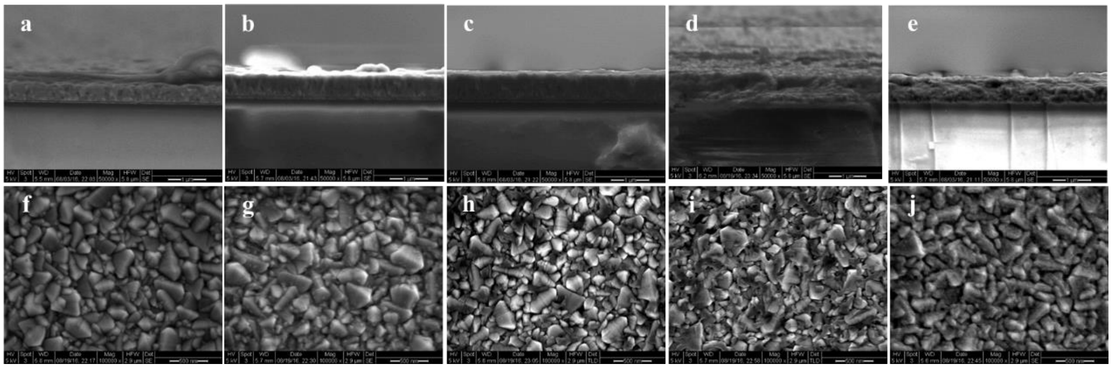

Supplementary Figure 4. SEM images of $BiVO_4$ film. Cross-sectional view (a to e) and top view (f to j) SEM images of $BiVO_4$ thin films on FTO made from different precursor concentrations. (a) and (f) is 0.01M, (b) and (g) is 0.02M, (c) and (h) is 0.04M, (d) and (i) is 0.08M, (e) and (j) is 0.08M but spin-coated twice, of the BiVO4 precursor same with the one described in Supplementary Figure 3. It can be seen that from (a) to (e) the sample thickness has no big change, but the surface coverage becomes higher with less FTO exposed.

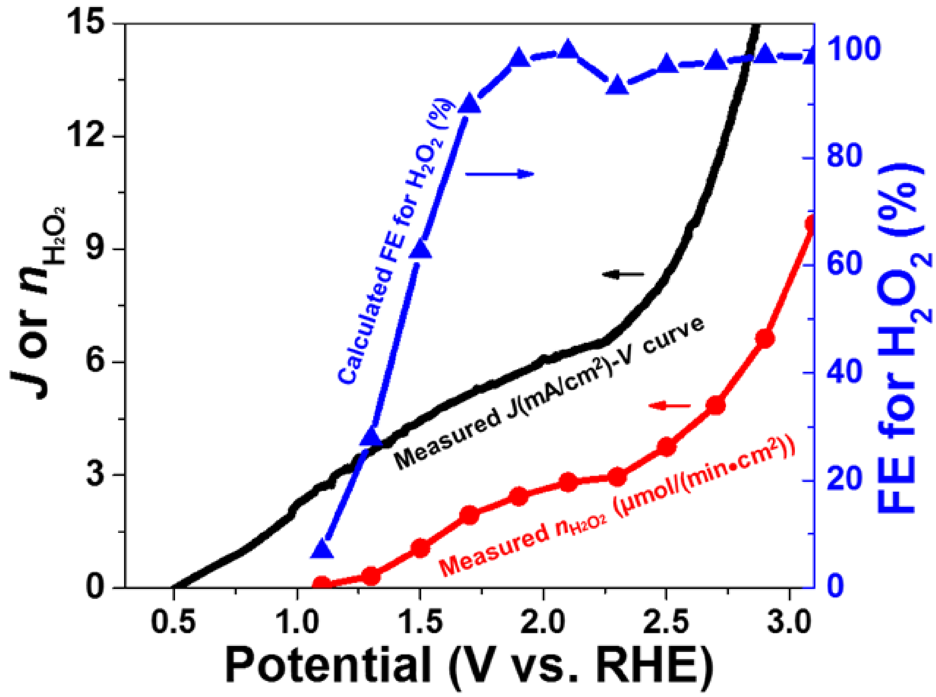

Supplementary Figure 5. *J-V*, H2O2 generation and calculated FE curves. The curves for the measured current-potential (*J-V*) and the measured H_2O_2 generation rate for $BiVO_4$ in bicarbonate solution under illumination. The calculated FE for H_2O_2 generation is shown as the blue curve, which is the one used in Figure 4 in the main text figures.

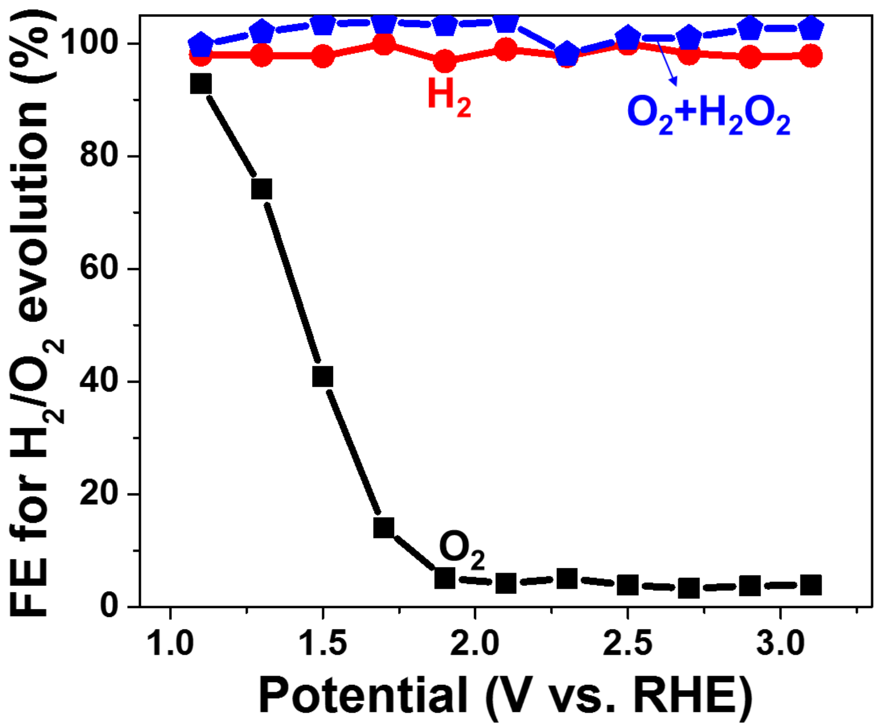

Supplementary Figure 6. The curves of FEs. These are for H_2 (red), O_2 (black) and $O_2+H_2O_2$ (blue) under varied applied potentials for the best condition, 9-layer $BiVO_4$ under illumination.

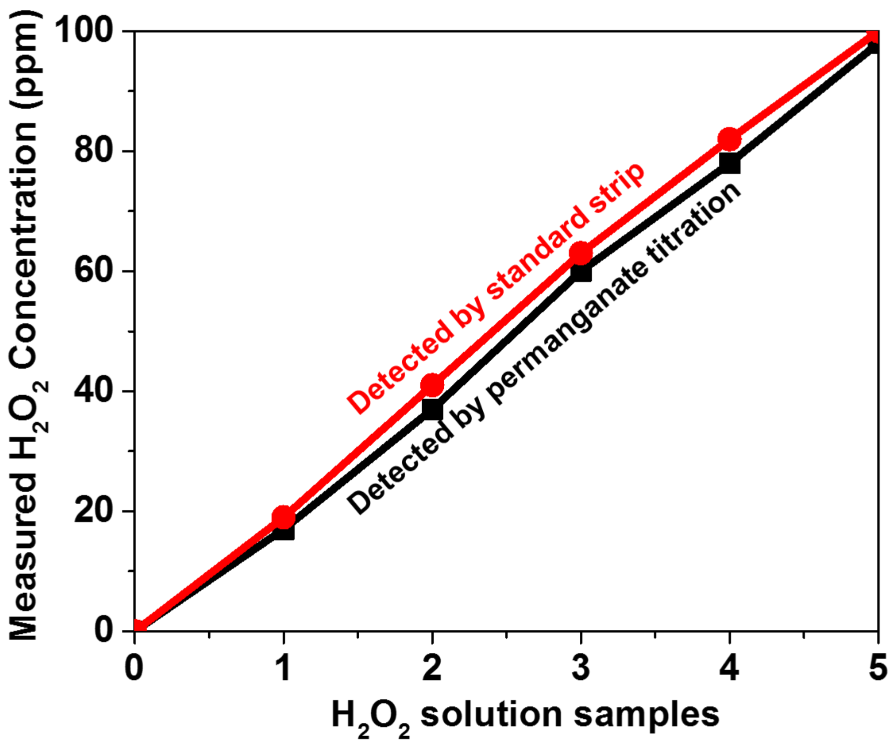

Supplementary Figure 7. H_2O_2 detection. H_2O_2 concentration detection for the five H_2O_2 solution samples with different degree of dilution from a same initial concentration 100ppm, by the standard strips (red) and the permanganate titration (black).

Supplementary Table 1. Free energies of adsorption. This table shows the free energies of adsorption for OH*, O* and OOH*, and overpotentials (η) for two-electron and four-electron water oxidation on surfaces of $BiVO_4$. Adsorption of OH*, O* and OOH* occurs on the bridge sites between Bi1 and Bi4 (14) as well as between Bi2 and Bi3 (23) shown in Supplementary Figure 1.

$BiVO_4$	(111) facet	
	14	23
ΔG_{OH}(eV)	1.95	2.07
ΔG_{O}(eV)	4.37	4.43
ΔG_{OOH}(eV)	4.83	5.10
2e-η^{OER} (V)	0.19	0.30
4e-η^{OER} (V)	1.19	1.13

Supplementary Table 2. Binding energies. This table shows the binding energies of OH^*, O^* and OOH^* adsorbates on the three different oxides, namely SnO_2, WO_3, TiO_2 plotted in Figure 1 of the main text.

**Oxide**	**ΔG_{OH} (eV)**	**ΔG_{O} (eV)**	**ΔG_{OOH} (eV)**
SnO_2[2]	2.02	5.00	5.31
WO_3[3]	1.66	3.82	4.71
TiO_2[2]	2.28	4.87	5.26

Supplementary Table 3. **Adsorption free energy**. Calculated adsorption free energy of OH* (ΔG_{OH}) and limiting potential (U_L) towards H_2O_2 formation in $BiVO_4$ (111) doped with different metals.

Doped Metals	ΔG_{OH} (eV)	U_L (V)
Ca_Bi4	2.41	2.41
Sr_Bi4	1.79	1.79
Ba_Bi4	2.23	2.23
Ir_Bi4	1.50	2.02
Ru_Bi4	1.68	1.84

Supplementary Table 4. Faraday Efficiency. The faraday efficiency (FE) dependency on sample preparation precondition for WO_3, SnO_2 and TiO_2 under their most appropriate applied bias (WO_3 @2.3V in 1M $NaHCO_3$ adjusted by H_2SO_4, pH6.0; SnO_2 @3.1V in 1M $NaHCO_3$, pH8.3; TiO_2 @3.3V in 1M $NaHCO_3$, pH 8.3; with all potentials *vs.* RHE). WO_3 was synthesized from the flame vapor deposition (FVD) with different growth time. SnO_2 and TiO_2 were synthesized from the sol-gel process (with different precursor concentration for $SnCl_2$, and different weight of TiO_2 powder in 5ml solvent, respectively), followed by spin-coating and annealing process as described in Methods section.

WO_3		SnO_2		TiO_2	
FVD time	FE	Precursor Concentration	FE	Precursor Concentration	FE
5min	9%	0.05M	37%	0.1g	7%
10min	46%	0.1M	51%	0.25g	18%
15min	31%	0.15M	25%	0.25g, 2-layer	13%

Supplementary Note 1:

Details of the calculated free energy of adsorptions:

We consider three intermediates in the oxygen evolution reaction (OER) namely, OH*, O* and OOH*. Catalytic activity of the material is determined by the binding energies of the reaction intermediates to the active sites of the catalyst.

To estimate the adsorption energies of different intermediates at zero potential and pH = 0 we calculate the reaction energies of each individual intermediate and correct them for zero point energy (ZPE) and entropy (TS) using the following equation: [1]

$$\Delta G = \Delta E + \Delta ZPE - T\Delta S$$

Additionally, we use the computational hydrogen electrode (CHE) model, which exploits that the chemical potential of a proton-electron pair is equal to gas-phase H_2 at standard conditions, and the electrode potential is taken into account by shifting the electron energy by $-eU$ where e and U are the elementary charge and the electrode potential, respectively. As the ground state of the O_2 is poorly described in DFT calculations we use gas phase H_2O and H_2 as reference states as they are readily treated in the DFT calculations. The entropy for H_2O is calculated at 0.035 bar which is the equilibrium pressure of H_2O at 300 K. The free energy of this state is therefore equal to that of liquid water.[1]

Supplementary Note 2:

Improving the activity of $BiVO_4$ by doping different metals

The activity of $BiVO_4$ can be further improved with different doped metals. We have investigated several dopants such as Ca, Sr, Ba, Ir, and Ru in the Bi4 site of Supplementary Figure 2. The results are given in Supplementary Table 3. Among the studied doped metals only Sr and Ru are shown to be capable of favorably changing the OH binding energy and increasing the activity towards H_2O_2 formation.

Supplementary Note 3:

Optimized conditions for metal oxides and selection of electrolyte

In this work, first we investigated the electrochemical properties of the four metal oxides without illumination. Since the electrochemical property of each oxide is affected by the amount of oxide coated on the substrate, from the beginning we identified the optimized coating condition of each oxide for H_2O_2 and then used the optimized condition for further comparison among these oxides. Taking $BiVO_4$ as an example. For $BiVO_4$ synthesis, different precursor concentration, and spin coating times was used. From Supplementary Figure 3, we got the information about the faraday efficiency for H_2O_2 generation, varying with the amounts of $BiVO_4$ coated on FTO. The results shown in this figure illustrates that when the loading of $BiVO_4$ on FTO is low, part of the FTO surface is exposed (Supplementary Figure 4), which has inferior property for H_2O_2 production. When the loading of $BiVO_4$ on FTO is high, the film resistance will be high and affect the performance. The optimal coating condition for $BiVO_4$ is one time spin coating by using the 0.08M precursor concentration. Similarly, for each oxide investigated, its loading on FTO was individually optimized to achieve high coverage on FTO while making film resistance as low as possible, as described in the main text.

In addition, since those oxides have different stable pH ranges, we further optimized the electrolyte for each oxide. For the case of $BiVO_4$, SnO_2 and TiO_2, $NaHCO_3$ is used as the electrolyte and the external biases were applied gradually from high to low to find out the onset potential for H_2O_2 generation (the method to define the onset potential for H_2O_2 generation is described in the Methods section). For the case of WO_3, both $NaHCO_3$ and H_2SO_4 have been examined. Pure $NaHCO_3$ has a pH value of around 7.9-8.3, which is too high for WO_3 to sustain its stability; and pure 0.5M H_2SO_4 with pH 0-1 is too low compared to the appropriate pH range of H_2O_2 production. Therefore, we modified the electrolyte by mixing $NaHCO_3$ with H_2SO_4 to adjust its pH. And finally biases were applied on it from high to low to get the onset value, similar to the methodology for $BiVO_4$, SnO_2, and TiO_2.

Supplementary References

1. Norskov, J. K. *et al.* Origin of the Overpotential for Oxygen Reduction at a Fuel Cell Cathode. (2004). doi:10.1021/JP047349J
2. Viswanathan, V., Hansen, H. A. & Nørskov, J. K. Selective Electrochemical Generation of Hydrogen Peroxide from Water Oxidation. *J. Phys. Chem. Lett.* **6,** 4224–8 (2015).
3. Siahrostami, S., Björketun, M. E., Strasser, P., Greeley, J. & Rossmeisl, J. Tandem cathode for proton exchange membrane fuel cells. *Phys. Chem. Chem. Phys.* **15,** 9326–34 (2013).
